# Effects of Dietary Calcium and Non-Phytate Phosphorus Interaction on Performance, Egg Quality and Lipid Metabolism of Aged Local Laying Hens

**DOI:** 10.3390/ani16193017

**Published:** 2026-09-25

**Authors:** Zhixun Yan, Qianqian Zhang, Weiyu Zhao, Cheng Chang, Yafang Zhao, Jian Zhang, Huagui Liu, Ailian Geng

**Affiliations:** Institute of Animal Husbandry and Veterinary Medicine, Beijing Academy of Agriculture and Forestry Sciences, Beijing 100097, China; yanzhixun2008@sina.com (Z.Y.);

**Keywords:** calcium, non-phytate phosphorus, egg quality, lipid metabolism, laying hen

## Abstract

This study aimed to investigate the effects of dietary calcium (Ca) and non-phytate phosphorus (NPP) levels alone and in interaction on performance, egg quality, lipid metabolism, antioxidant capacity and follicular development in aged local laying hens. A total of 900 44-week-old *Beijing You Chicken* (BYC) laying hens with an average body weight of 2.13 ± 0.12 kg were randomly allocated to nine groups (100 hens/group, four replicates of 25 hens). A 3 × 3 factorial design was used with Ca at 3.0%, 3.4%, 3.8% and NPP at 0.31%, 0.36%, 0.41%, respectively. After 1 week of adaptation, the trial lasted from 45 to 58 weeks of age. The results showed that dietary Ca and the NPP level alone did not affect the egg-laying rate of BYCs during 45–58 weeks (*p* > 0.05), but their interaction affected egg mass (EM, *p* = 0.049). The group with 3.0% Ca and 0.31% NPP exhibited the highest EM (0.22 kg). At 52 weeks of age, the dietary Ca level affected yolk color (YC): 3.4% Ca and 3.6% Ca increased YC (*p* < 0.05). Dietary 0.41% NPP decreased YC (*p* < 0.05). The Ca and NPP interaction affected the albumen height (AH), Haugh unit (HU) and YC (*p* < 0.05). The group with 3.0% Ca and 0.41% NPP had the highest AH (5.14) and HU (73.46). At 58 weeks of age, 3.4% Ca indicated the lowest YC (*p* < 0.05). Moreover, 0.36% NPP had the lowest YC and the highest RYW (*p* < 0.05). Significant Ca × NPP interactions were observed for eggshell thickness (EST), AH, HU, YC, and egg grade (EG) (*p* < 0.05). The group with 3.0% Ca and 0.41% NPP exhibited the highest AH, HU, YC, and EG. The Ca × NPP interaction affected high-density lipoprotein cholesterol (HDL-C) and low-density lipoprotein cholesterol (LDL-C) content (*p* < 0.05). Dietary 0.41% NPP increased serum malondialdehyde (MDA) content (*p* = 0.020). The group with 3.0% Ca and 0.41% NPP had the lowest serum LDL-C (*p* < 0.05). Dietary Ca, the NPP level alone, and their interaction might not affect the number of large yellow follicles and graded follicles at 58 weeks of age (*p* > 0.05). The 0.36% NPP and 0.41% NPP increased the number of small yellow follicles (*p* = 0.021). The group with 3.4% Ca and 0.36% NPP had the highest number of small yellow follicles (25.75 ± 4.34, *p* = 0.033). In summary, the dietary Ca and NPP interaction, rather than Ca or the NPP level alone, contributed to egg mass, egg quality, lipid metabolism and small yellow follicles in the late laying period. An excessive NPP level (0.41%) may not bring additional benefits to the late laying hens’ performance and could lead to increased peroxidation in the aging local laying hens.

## Simple Summary

The late laying period accounts for approximately half of the laying period, when the laying hens’ performance declines and egg quality deteriorates, accompanied by altered lipid metabolism and increased oxidative stress. Calcium and phosphorus are the two most important essential minerals, and they are the third and fourth most expensive components in feed costs. This study aimed to investigate the effects of dietary calcium and non-phytate phosphorus levels alone and in interaction on performance, egg quality, lipid metabolism, antioxidant capacity and follicular development in aged local laying hens. The results showed that dietary calcium and the non-phytate phosphorus level alone did not affect the egg-laying rate of *Beijing You Chicken laying hens* during 45–58 weeks, but their interaction affected egg mass. Significant calcium and non-phytate phosphorus interactions were observed for eggshell thickness, albumen height, Haugh unit, yolk color, and egg grade at 58 weeks of age. Dietary 0.41% non-phytate phosphorus increased the serum malondialdehyde content. The calcium and non-phytate phosphorus interaction affected the high-density lipoprotein cholesterol and low-density lipoprotein cholesterol content. In summary, dietary calcium and non-phytate phosphorus interaction, rather than calcium or the non-phytate phosphorus level alone, contributed to egg mass, egg quality, lipid metabolism and small yellow follicles in the late laying period.

## 1. Introduction

China is one of the major producers and consumers of poultry eggs in the world. The chicken egg production reached 35.63 million tons in 2023, ranking first in the world [1]. The egg-laying process of ordinary hens usually can be divided into three periods: the pre-laying period, the peak laying period, and the late laying period. When the egg-laying rate drops to 80% of the peak laying period, the laying hen enters the late laying period (about 400 d of age for high-yield breeds and about 300 d of age for low-yield breeds) [2]. In a 700-d production cycle, the late laying period accounts for approximately half of the laying period. As laying hens progress into the late laying period, production performance declines and egg quality deteriorates [3], accompanied by metabolic challenges such as altered lipid metabolism and increased oxidative stress, decreased bone mineralization and increased susceptibility to metabolic disorders [4]. The nutritional strategies that can promote health and maintain productivity in the late laying period will become increasingly important.

Calcium (Ca) and phosphorus (P) are the two most important essential minerals involved in eggshell formation and multiple physiological processes, including neuromuscular function and energy metabolism [5,6,7], and they are the third and fourth most expensive nutritional components in feed costs after energy and crude protein [3]. Non-phytate phosphorus (NPP) can provide a more precise estimate of the P requirement for poultry [8]. The egg production of hens aged 26 to 36 weeks increased as dietary NPP levels rose from 0.17% to 0.34% [9]. Insufficient Ca supply can compromise egg production and markedly lower eggshell quality, resulting in more soft-shell eggs and broken eggs. Excessive dietary Ca and P may reduce nutrient utilization and negatively affect performance [10,11]. Pelicia et al. [12] found that the egg-laying rate decreased when Hy-line brown laying hens were fed 4.0% Ca compared with 3.5% Ca in their diet. Therefore, maintaining appropriate dietary Ca and NPP levels is critical for laying hens, particularly during the late laying period, when performance declines and egg quality deteriorates.

Studies on aged hens have shown that Ca and NPP nutrition interacts with physiological remodeling of mineral homeostasis and can affect eggshell quality and skeletal status [7]. The responses to changes in NPP in older hens are not always consistent with the results of some studies, which report improvements in performance or eggshell quality under specific feeding strategies [13], while another study has observed limited effects on production but changes in mineral-related traits [14]. These findings highlight the need to define breed and stage-specific Ca and NPP recommendations under practical production conditions.

*Beijing You Chicken* (BYC) is an important local breed in China, valued for its meat and egg quality; its egg output is lower than that of commercial high-yield strains (e.g., *Roman Brown, Hy-line Brown*), with 150–170 eggs per year [15]. Due to its low egg output, dietary Ca and NPP requirements of BYC may differ from those of high-yield strains. The egg production and egg quality decline rapidly during the late laying period (after 44 weeks), which causes economic losses to the breeding industry. Therefore, clarifying the key nutritional requirements at this stage holds significant practical significance for production. Our previous works have evaluated different dietary Ca and NPP levels of BYC during the growing period and peak laying period, indicating that dietary Ca and NPP levels influence production and serum biochemical parameters, and some indices were improved by the interaction of Ca and NPP [16,17]. This study aims to further investigate the effects of dietary Ca and NPP levels alone and in interaction on performance, egg quality, lipid metabolism, antioxidant capability and follicular development in aged BYC laying hens, providing a reference for precise mineral requirements of chickens at different periods.

## 2. Materials and Methods

### 2.1. Experimental Design and Birds

The experiment was conducted at the BYC demonstration farm in Shunyi district, Beijing. A 3 × 3 factorial design was used, with 3 dietary Ca levels (3.0%, 3.4%, and 3.8%) and 3 dietary NPP levels (0.31%, 0.36%, and 0.41%). A total of 900 44-week-old BYC laying hens with a similar body weight of 2.13 ± 0.12 kg were randomly allocated to 9 groups (100 hens/group, 4 replicates of 25 hens). The hens were fed the peak laying diet from 22 to 43 weeks and fed the late laying diet from 44 weeks of age. The composition and nutrient levels of the basal diet are presented in Table 1. The diets were formulated using nutrient-matching software, and the key nutrient levels were balanced across the diets. Dietary Ca and NPP levels were adjusted in accordance with Technical code of practice for feeding and management of *Beijing You Chicken* [18].

### 2.2. Feeding and Management

The experiment began at 44 weeks of age, including a 1 week adaptation period. Subsequently, a formal feeding trial was conducted from 45-to-58 weeks of age. The hens were housed in a three-tier A-type cage system, with each replicate evenly distributed across each tier. The chickens had free access to feed and water and were managed according to standard commercial practices. Throughout the experiment, a 16 h light (6:00–22:00) and 8 h dark schedule was applied.

### 2.3. Performance

During the experiment, the number of hens, egg number and weight were recorded daily for each replicate. Egg-laying rate and egg mass (EM) were calculated during 45–58 weeks (hen/week).

### 2.4. Egg Quality

At 52 and 58 weeks of age, qualified eggs were randomly selected from each replicate group, yielding 7, 7, 8, and 8 eggs per replicate respectively, totaling 30 eggs per treatment group. Egg weight (EW), albumen height (AH), Haugh unit (HU, a key parameter for assessing egg freshness, which is derived from the height of the thick albumen and total egg weight via a standardized formula), yolk color (YC), egg grade (EG), eggshell strength (ESS), eggshell thickness (EST), eggshell color (ESC), egg shape index (ESI), relative yolk weight (RYW), relative eggshell weight (RESW), and relative albumen weight (RAW) were measured and calculated within 24 h. EW, AH, HU, YC, and EG were determined by using the Egg Multi Tester (Model EMT-7300, Robotmation Co., Ltd., Tokyo, Japan). Egg grade included AA, A, B, and C, represented as a score of 3, 2, 1, or 0, respectively. ESS was determined by using an Egg Force Reader (Orka Food Technology Ltd., Herzliya, Israel). EST was determined by using an Echo-meter (Model ETG-1061A, Karl Deutsch, Wuppertal, Germany). ESC was determined by using a TSS OCR-PS (Technical Services and Supplies, York, UK). ESI was determined by using an Egg Shape Index Tester (FHK, Fujihira Industry Co., Ltd., Tokyo, Japan). The yolk and albumen were separated and weighed, and RYW, RAW, and RESW were calculated accordingly.

### 2.5. Serum Biochemical Indicators and Lipid Metabolism

At the end of 58 weeks, after overnight feed withdrawal, 5 mL of blood samples was collected from the wing vein of 3 hens per replicate. The samples were centrifuged at 3000× *g* for 10 min at 4 °C, and the serum was stored at −80 °C until analysis. Serum Ca, P, triglycerides (TGs), total cholesterol (TC), glucose (Glu), high-density lipoprotein cholesterol (HDL-C), low-density lipoprotein cholesterol (LDL-C), lactate dehydrogenase (LDH), malondialdehyde (MDA), and total antioxidant capacity (T-AOC) were measured using commercial kits (Nanjing Jiancheng Bioengineering Institute, Nanjing, China). Absorbance was determined using a spectrophotometer (Evolution 60, Thermo Fisher Scientific, Shanghai, China) or a microplate reader (Multiskan FC, Thermo Fisher Scientific, Shanghai, China). The catalogue numbers and assay methods for the commercial kits were seen in Supplementary Materials (Table S1).

### 2.6. Follicular Development

At the end of 58 weeks, one hen was randomly selected from each replicate and 4 hens from each group. Cervical dislocation was performed, followed by dissection to observe the follicle size and quantity. Follicles were isolated and the follicle diameter was measured using an electronic vernier caliper. Follicles were classified into graded follicles (≥12 mm), large yellow follicles (9~11 mm), and small yellow follicles (6~8 mm) [17].

### 2.7. Data Analysis

Data were analyzed by using the General Linear Model (GLM) procedure in SPSS 25.0 software for Windows (SPSS Inc., Chicago, IL, USA). The effects of the dietary Ca level (3.0%, 3.4%, and 3.8%) and NPP level (0.31%, 0.36%, and 0.41%) were analyzed as a 3 × 3 factorial design. The statistical model included Ca, NPP and their interaction (Ca × NPP) as fixed effects, and the replicate as a random factor. The linear model was:

Y_ijk_ = μ + Ca_i_ + NPP_j_ + (Ca × NPP)_ij_ + R_k_ + ε_ijk_

where Y_ijk_ is the observed response; μ is the overall mean; Ca_i_ and NPP_j_ are the main effects of the i-th and j-th levels; (Ca × NPP)_ij_ is their interaction; R_k_ is the random effect of the k-th replicate (R_k_~N (0,σ^2^_R_); and ε_ijk_ is the residual error.

Prior to ANOVA, the assumptions of normality and homogeneity of variances were assessed. Percentage data (e.g., laying rate, relative yolk weight) were arcsine-transformed to improve normality. Descriptive statistics in tables are presented as untransformed means, while the corresponding *p*-values are derived from transformed data. Normality was checked using the Shapiro–Wilk test, and the homogeneity of variances was verified using Levene’s test; both assumptions were met (*p* > 0.05) for all untransformed response variables, confirming the appropriateness of the ANOVA.

For egg quality and serum parameters, individual eggs and hens were used in the GLM for analysis. The nested design was indicated by coding the replicate effect as replicate(treatment), and a variance components covariance structure was specified for the random replicate effect.

When the Ca × NPP interaction was significant, simple effects were examined among the nine treatment groups. When the interaction was not significant, main effects were interpreted. Treatment means were separated using Duncan’s multiple range test at α = 0.05. Statistical significance was declared at *p* < 0.05, and a tendency was considered at 0.05 < *p* < 0.10; *p* < 0.001 was considered highly significant.

The performance and follicular development data are presented as the mean and standard deviation. The egg quality and serum indices data are presented as the mean and standard error of mean.

## 3. Results

### 3.1. Performance During 45–58 Weeks

As shown in Table 2, the dietary Ca and NPP level alone did not affect the performance of BYCs during 45–58 weeks (*p* > 0.05), but their interaction affected EM (*p* = 0.049); the group with 3.0% Ca and 0.31% NPP exhibited the highest EM (0.22 kg). The interaction of Ca and NPP had the tendency to affect the egg-laying rate; the group with 3.0% Ca and 0.31% NPP had the highest egg-laying rate (66.08%, *p* = 0.058).

### 3.2. Egg Quality at 52 Weeks of Age

Table 3 shows that the dietary Ca level affected YC. Compared to 3.0% Ca, dietary 3.4% Ca and 3.8% Ca increased YC (*p* < 0.05). Dietary 0.41% NPP decreased YC compared to 0.31% NPP and 0.36% NPP (*p* < 0.05). The Ca × NPP interaction affected AH (*p* = 0.012), HU (*p* = 0.023) and YC (*p* < 0.001). The group with 3.0% Ca and 0.41% NPP had the highest AH (5.14) and HU (73.46). EST had the tendency of being affected by Ca × NPP interaction (*p* = 0.060).

### 3.3. Egg Quality at 58 Weeks of Age

Table 4 shows that the dietary Ca level affected YC, 3.4% Ca indicating the lowest YC (*p* < 0.05). Dietary NPP affected YC and RYW; 0.36% NPP had the lowest YC (*p* < 0.05) and the highest RYW (*p* < 0.05). Significant Ca × NPP interactions were observed for EST (*p* = 0.049), AH (*p* = 0.005), HU (*p* = 0.006), YC (*p* < 0.05), and EG (*p* = 0.001). The group with 3.0% Ca and 0.41% NPP exhibited the highest AH, HU, YC, and EG at 58 weeks of age.

### 3.4. Serum Lipid Metabolism and Antioxidant Capacity at 58 Weeks of Age

Table 5 indicates that serum Ca content was not affected by dietary Ca, the NPP level and their interaction (*p* > 0.05). Dietary 3.4% Ca had the tendency to decrease serum *p* content (*p* = 0.069), dietary 0.36% NPP had the tendency to decrease TG content (*p* = 0.074), and dietary 0.36% NPP and 0.41% NPP had the tendency to increase Glu content (*p* = 0.098) and HDL-C content (*p* = 0.051). The Ca × NPP interaction affected HDL-C and LDL-C contents (*p* = 0.023, *p* = 0.007). The group with 3.0% Ca and 0.41% NPP had the lowest serum LDL-C (*p* < 0.05). Dietary 0.41% NPP increased serum MDA content when compared with 0.31% NPP and 0.36% NPP (*p* = 0.020). T-AOC and LDH content were not affected by Ca, the NPP level alone, and their interaction (*p* > 0.05).

### 3.5. Follicular Development at 58 Weeks of Age

Table 6 indicates that dietary Ca, the NPP level, and their interaction might not affect the number of large yellow follicles and graded follicles (*p* > 0.05). An amount of 0.36% NPP and 0.41% NPP increased the number of small yellow follicles (*p* = 0.021). The interaction of Ca and NPP affected the number of small yellow follicles (*p* = 0.033); the group with 3.4% Ca and 0.36% NPP had the highest number of small yellow follicles (25.75 ± 4.34).

## 4. Discussion

The responses of performance to dietary Ca and the NPP level vary among different studies, reflecting differences in breed, age, basal diet, and Ca and NPP levels. Reducing NPP to an extremely low level may inhibit egg production in some strains [22], while moderately adjusting NPP within a practical range may have a limited effect on performance when the diet meets the required level [15,16]. In the present study, neither dietary Ca nor the NPP level affected the egg-laying rate during 45–58 weeks, which is consistent with our previous findings [17]. It is possible that within the ranges (Ca 3.0–3.8% and NPP 0.31–0.41%), the diets provided adequate minerals to maintain production, and short-term performance responses to single-factor changes were limited. A significant interaction of Ca and NPP was observed for egg mass, indicating that the dietary Ca/NPP ratio, rather than Ca or NPP alone, contributed to improvement in the total egg weight.

It was reported that the egg-laying rate increased when the dietary Ca level increased from 3.5% to 4.0% in *H&N Brown* egg layers (60 weeks old) [23], but the effect of dietary NPP increase is not clear. In the present study, a higher dietary NPP level (0.41%) may not bring additional benefits to the late local laying hens’ performance. This might be due to the reduced P requirement caused by their low egg production, as well as the age-related changes in the intestinal absorption efficiency of the hens [7].

Ca is directly related to eggshell thickness and strength. A typical eggshell contains approximately 2.3–2.6 g Ca. The P accounts for a relatively small proportion of the eggshell, but it influences eggshell quality indirectly through its interaction with Ca metabolism and mineral balance. The Ca and P supply and their ratio affect both eggshell formation and bone integrity, especially in aged laying hens; mineral nutrition is closely linked to egg quality and hen health in the late laying period [3]. Inappropriate Ca/P ratios can impair eggshell traits and bone mineralization [24,25]. However, eggshell responses to dietary Ca can be variable. Cufadar et al. [26] reported no significant changes in eggshell weight or thickness across Ca concentrations, while eggshell strength was affected. Zhang [27] reported that dietary 3.5% Ca significantly improved eggshell strength in late laying hens when compared with dietary 3.0% Ca. In the present study, the dietary Ca or NPP level alone did not affect eggshell strength at either 52 or 58 weeks of age, while Ca and NPP interaction affected eggshell thickness at 58 weeks of age, indicating that the present dietary Ca and NPP supply was sufficient to maintain eggshell formation and strength, while eggshell thickness was more responsive to Ca and NPP interaction. The Ca and NPP interactions also affected the albumen height, Haugh unit and yolk color at 52 weeks and 58 weeks of age, indicating that internal egg quality was affected by the Ca/NPP ratio. Notably, 3.0% Ca and 0.41% NPP, a Ca/NPP ratio of 7.32:1, may better support internal egg quality in the late laying period.

Ca and NPP participate broadly in metabolic processes linked to protein utilization and turnover, which may indirectly influence the albumen structure and quality [28]. The Ca/NPP ratio may influence yolk pigmentation, although the response was not uniform across the treatments. Pelicia et al. [29] observed a linear increase in yolk color in *Hisex Brown* layers aged 90 weeks with increasing dietary Ca, while yolk color was not affected by the NPP level; Eltahan et al. [13] reported no effect of the NPP level on yolk color in *Hy-Line Brown* hens at 76 and 80 weeks. The present study indicated that dietary 3.4% Ca and 3.8% Ca increased the yolk color at 52 weeks of age, and 0.41% NPP indicated the lowest YC at 58 weeks of age, which was in line with our previous report that dietary 3.6% Ca deepened the yolk color and the dietary NPP level affected YC in the peak laying period [17]. The minor differences in wheat bran and soybean oil in the experimental diet may have some potential confounding effects on the yolk color. When the basal Ca and NPP supply is adequate for shell formation, optimizing the Ca/NPP ratio appears more relevant for maintaining the internal egg quality of laying hens.

Serum biochemical indices provide an integrated reflection of mineral homeostasis and metabolic status in laying hens. Serum Ca and P concentrations are tightly regulated by intestinal absorption, renal excretion, and bone mobilization, forming a dynamic buffering system that maintains physiological stability under a wide range of dietary conditions. Serum mineral responses to dietary Ca and P levels vary with age, physiological stage, and breeds [30], and serum Ca and P remain relatively stable despite dietary adjustments [31,32]. In the present study, dietary 3.4% Ca had the tendency to decrease serum P content, which contrasts with our observations in growing pullets, where dietary Ca or NPP was reported to influence serum mineral concentrations [14], suggesting that the late laying hens may possess a stronger capacity to maintain mineral homeostasis, reflecting an increased dependence on bone-derived Ca and more effective endocrine regulation of Ca and P metabolism in the late laying period. This needs to be verified by measurements of bone mineralization, vitamin D content, etc. in the future.

Serum HDL-C prevents atherosclerosis by transporting cholesterol to the liver for metabolism, while LDL-C causes atherosclerosis by depositing cholesterol on the arterial walls [33]. Therefore, higher HDL-C levels and lower LDL-C levels are beneficial to health. A dietary 4% Ca level significantly reduced serum triglycerides and low-density lipoprotein in aged *H&N Brown* layers compared with a 3.5% Ca level; the triglyceride/high-density lipoprotein ratio decreased in both the 4 and 4.5% Ca-level diets [23]. The group of laying hens with 3.6% Ca and 0.15% NPP showed reduced plasma phospholipid levels, yet increased plasma TG, free fatty acid levels, and hepatic lipoprotein lipase activity, indicating that an inappropriate Ca/NPP ratio can lead to disturbed lipid metabolism [34]. The present study found that the group with 3.0% Ca and 0.41% NPP had the lowest serum LDL-C level and a non-significant HDL-C level, suggesting that 3.0% Ca and 0.41% NPP can help reduce the risk of atherosclerosis.

Serum MDA is an indicator of lipid peroxidation. Attia et al. [23] found that dietary Ca levels (3.5%, 4.0%, 4.5%) had no effects on MDA content in H&N Brown layers at 72 weeks of age. Our previous study showed that serum MDA increased in the 0.43% NPP group, but it did not reach a significant level [17]. In the present study, the dietary Ca level had no effects on MDA content, but dietary 0.41% NPP exhibited the highest MDA. This can be further verified by more oxidative stress markers at the tissue level.

Our previous work showed associations between the dietary Ca/NPP ratio and metabolic enzyme activity [17], supporting the view that Ca and NPP interaction can influence the overall metabolic status beyond the internal mineral homeostasis. The present study indicates that excessive dietary NPP may impose oxidative stress in the late laying hens, potentially through altered lipid metabolism or an increased metabolic load, highlighting the importance of avoiding unnecessary NPP and inappropriate Ca/NPP in the late laying period.

Follicular development in laying hens is affected by the intrinsic endocrine regulation and extrinsic nutritional status [35]. Ca and P function not only as structural minerals but also as modulators of cellular signaling and metabolic processes. Ca-dependent signaling pathways are essential for granulosa cell proliferation and steroidogenesis, whereas phosphorus availability supports energy metabolism and nucleotide synthesis required for follicular growth and maintenance [36]. We previously found that dietary 0.33% NPP and 0.43% NPP increased the number of small yellow follicles of BYC at 41 weeks of age [17]. In the present study, we further found that 0.36% NPP and 0.41% NPP increased the number of small yellow follicles of BYC at 58 weeks of age. The interaction of Ca and NPP also affected the number of small yellow follicles. This is a very interesting result, which may help find a way to modulate the pre-graded follicular development through modulating dietary NPP levels in the late laying period. In the future, it is necessary to further confirm this under the condition of increasing the sample size.

## 5. Conclusions

The present study suggested that dietary Ca and NPP interaction, rather than the Ca or NPP level alone, contributed to egg mass, egg quality, lipid metabolism and small yellow follicles in the late laying period. Dietary 3.0% Ca and 0.31% NPP, corresponding to a Ca/NPP ratio of 9.68:1, benefited egg mass; dietary 3.0% Ca and 0.41% NPP, corresponding to a Ca/NPP ratio of 7.32:1, benefited egg quality and lipid metabolism, while dietary 3.4% Ca and 0.36% NPP (Ca/NPP ratio of 9.44:1) benefited small yellow follicles. An excessive NPP level (0.41%) may not bring additional benefits to late laying hens’ performance and could lead to increased peroxidation in aging local laying hens. Future studies are needed to clarify the effects of excessive NPP, and the related mechanism by which Ca and NPP interaction regulates lipid metabolism and follicular development, in order to optimize their application in precise mineral nutrition.

## Figures and Tables

**Table 1:** Composition and nutrient levels of basal diets (air-dry basis).

Items (%)	1	2	3	4	5	6	7	8	9
Ca	3.0	3.4	3.8	3.0	3.4	3.8	3.0	3.4	3.8
NPP	0.31	0.31	0.31	0.36	0.36	0.36	0.41	0.41	0.41
Ca/NPP	9.68	10.97	12.26	8.33	9.44	10.55	7.32	8.29	9.27
Composition									
Corn	68.85	67.90	65.90	69.00	67.84	65.62	69.15	67.54	65.33
Wheat bran	1.00	0	0	0.60	0	0	0.13	0	0
Soybean meal 46	20.50	20.80	20.75	20.57	20.42	20.78	20.65	20.48	20.85
Corn gluten meal 60	0	0.10	0.40	0	0.40	0.40	0	0.40	0.40
Soybean oil	0	0.40	1.05	0	0.40	1.15	0	0.50	1.23
Dicalcium phosphate	0.89	0.90	0.90	1.13	1.14	1.15	1.37	1.38	1.39
Limestone powder 36	7.76	8.90	10.00	7.70	8.80	9.90	7.70	8.70	9.80
Premix ^1^	1.00	1.00	1.00	1.00	1.00	1.00	1.00	1.00	1.00
Total	100.00	100.00	100.00	100.00	100.00	100.00	100.00	100.00	100.00
Nutrient levels									
ME/(MJ/kg) ^2^	11.500	11.503	11.505	11.505	11.503	11.505	11.507	11.504	11.502
CP/(%)	15.045	15.012	15.013	15.026	15.012	15.005	15.001	15.017	15.014
Lysine	0.778	0.779	0.776	0.778	0.771	0.777	0.777	0.772	0.778
Methionine	0.393	0.393	0.395	0.393	0.396	0.395	0.393	0.395	0.395
Cystine	0.256	0.255	0.254	0.256	0.255	0.254	0.255	0.255	0.254
Methionine + Cystine	0.650	0.648	0.649	0.649	0.650	0.649	0.648	0.650	0.649
Tryptophan	0.177	0.176	0.176	0.177	0.175	0.176	0.176	0.175	0.176
Ca ^3^	3.003	3.414	3.810	3.015	3.411	3.809	3.048	3.409	3.807
Total P	0.516	0.509	0.505	0.564	0.558	0.556	0.611	0.608	0.607
NPP ^4^	0.311	0.310	0.309	0.361	0.360	0.361	0.410	0.411	0.411

^1^ The premix provided the following per kg of the diet: VA 7.200 IU, VD 3360 IU, VE 20 IU, VK 2.9 mg, thiamine 2.4 mg, riboflavin 6.2 mg, calcium pantothenate 13 mg, niacin 36 mg, pyridoxine 4.3 mg, Biotin 0.24 mg, folic acid 1.5 mg, VB12 0.032 mg, choline 800 mg, Mn 100 mg, I 1.8 mg, Fe 100 mg, Cu 8 mg, Zn 90 mg, Se 0.30 mg. ^2^ ME = metabolizable energy; CP = crude protein; Ca = Calcium; NPP = non-phytate phosphorus. ^3^ Ca and NPP are measured values. The determination of dietary Ca follows the Chinese national standard GB/T6436-2018 [19], and the determination of dietary NPP follows the Chinese national standard GB/T6437-2018 [20]. The rest of the nutrient levels were calculated from the data provided by the Feed Database in China (2013) [21]. ^4^ The group with 3.4% Ca and 0.36% NPP was the control group, representing the farm’s commercial diet, to provide a practical reference.

**Table 2:** Effects of dietary calcium and non-phytate phosphorus levels on performance of *Beijing You Chicken* during 45–58 weeks (*n* = 4).

Items		Egg-Laying Rate (%)	EM (kg)
Ca (%)	NPP (%)		
3.0	0.31	66.08 ± 0.06	0.22 ± 0.03 ^b^
3.4	0.31	56.31 ± 0.09	0.19 ± 0.03 ^a^
3.8	0.31	58.85 ± 0.08	0.20 ± 0.02 ^ab^
3.0	0.36	58.92 ± 0.08	0.20 ± 0.02 ^ab^
3.4	0.36	56.92 ± 0.09	0.20 ± 0.03 ^ab^
3.8	0.36	59.54 ± 0.08	0.21 ± 0.03 ^ab^
3.0	0.41	55.69 ± 0.10	0.19 ± 0.02 ^a^
3.4	0.41	59.54 ± 0.08	0.20 ± 0.03 ^ab^
3.8	0.41	58.15 ± 0.09	0.20 ± 0.03 ^ab^
Ca (%)	3.0	60.23	0.20
	3.4	57.59	0.20
	3.8	58.85	0.20
	*p*-value	0.373	0.269
NPP (%)	0.31	60.41	0.20
	0.36	58.46	0.20
	0.41	57.79	0.20
	*p*-value	0.374	0.335
Ca × NPP	*p*-value	0.058	0.049

Note: Ca = calcium; NPP = non-phytate phosphorus; EM = egg mass. Data in the same column without letters or the same letters superscripts mean no significant difference (*p* > 0.05); different letter superscripts mean significant difference (*p* < 0.05 as statistically significant, and 0.05 < *p* < 0.10 as having a trend, *p* < 0.001 as highly significant); the same below.

**Table 3:** Effects of dietary calcium and non-phytate phosphorus levels on egg quality of 52-week-old *Beijing You Chicken* (*n* = 30).

Items		EW (g)	ESS (kg/cm^2^)	EST (mm)	ESC	AH (mm)	HU	YC	EG	ESI	RYW (%)	RESW (%)	RAW/%
Ca (%)	NPP (%)												
3.0	0.31	47.97	3.61	0.32	52.01	4.54 ^a^	68.46 ^ab^	11.54 ^b^	2.30	1.36	31.84	12.90	55.26
3.4	0.31	48.28	3.59	0.33	50.29	5.01 ^b^	72.17 ^ab^	11.31 ^ab^	2.40	1.33	31.17	13.28	55.55
3.8	0.31	49.49	3.42	0.32	51.07	4.45 ^a^	67.88 ^ab^	12.49 ^d^	2.27	1.34	31.27	13.49	55.24
3.0	0.36	48.95	3.71	0.32	52.04	4.72 ^ab^	69.23 ^ab^	11.32 ^ab^	2.30	1.34	31.87	13.67	54.46
3.4	0.36	49.05	3.36	0.31	50.27	4.91 ^ab^	70.86 ^ab^	11.39 ^b^	2.37	1.34	32.24	13.17	54.59
3.8	0.36	48.69	3.61	0.33	51.50	4.84 ^ab^	71.84 ^ab^	12.35 ^dc^	2.50	1.33	31.21	13.81	54.98
3.0	0.41	48.74	3.47	0.32	50.85	5.14 ^b^	73.46 ^b^	10.97 ^a^	2.58	1.36	32.12	13.68	54.20
3.4	0.41	49.82	3.59	0.32	50.85	4.25 ^a^	65.72 ^a^	12.09 ^c^	2.03	1.36	31.96	13.49	54.55
3.8	0.41	48.89	3.53	0.31	50.83	5.09 ^b^	73.22 ^b^	11.43 ^b^	2.41	1.36	31.55	13.20	55.25
SEM		0.239	0.046	0.002	0.392	0.077	0.705	0.044	0.048	0.004	0.001	0.001	0.265
Ca (%)	3.0	48.55	3.60	0.32	51.63	4.80	70.39	11.28 ^a^	2.39	1.35	31.93	13.42	54.62
	3.4	49.05	3.51	0.32	50.47	4.72	69.58	11.60 ^b^	2.27	1.34	31.40	13.35	54.90
	3.8	49.02	3.52	0.32	51.13	4.79	70.98	12.09 ^b^	2.39	1.34	30.94	13.50	55.16
	*p*-value	0.633	0.726	0.915	0.479	0.90	0.708	<0.001	0.462	0.474	0.213	0.476	0.136
NPP (%)	0.31	48.58	3.54	0.32	51.12	4.67	69.50	11.78 ^b^	2.32	1.34 ^ab^	31.40	13.27	55.35
	0.36	48.90	3.56	0.32	51.27	4.82	70.65	11.69 ^b^	2.39	1.33 ^a^	31.08	13.55	54.68
	0.41	49.15	3.53	0.32	50.84	4.83	70.80	11.49 ^a^	2.34	1.36 ^b^	31.84	13.50	54.67
	*p*-value	0.613	0.972	0.852	0.903	0.615	0.706	0.025	0.841	0.047	0.413	0.199	0.852
Ca × NPP	*p*-value	0.528	0.393	0.060	0.941	0.012	0.023	<0.001	0.125	0.543	0.706	0.138	0.712

Note: Ca = calcium; NPP = non-phytate phosphorus; EW = egg weight; ESS = eggshell strength; EST = eggshell thickness; AH = albumen height; HU = Haugh unit; YC = yolk color; EG = egg grade; ESI = egg shape index; RYW = relative yolk weight; RESW = relative eggshell weight; RAW = relative albumen weight.

**Table 4:** Effects of dietary calcium and non-phytate phosphorus levels on egg quality of 58-week-old *Beijing You Chicken* (*n* = 30).

Items		EW (g)	ESS (kg/cm^2^)	EST (mm)	ESC	AH (mm)	HU	YC	EG	ESI	RYW (%)	RESW (%)	RAW (%)
Ca (%)	NPP (%)												
3.0	0.31	50.44	3.63	0.32 ^c^	55.37	5.04 ^a^	71.98 ^ab^	11.77 ^d^	2.57 ^bc^	1.31	31.43	12.43	56.14
3.4	0.31	50.70	3.47	0.30 ^bc^	53.40	4.61 ^a^	68.08 ^a^	11.13 ^bc^	1.95 ^a^	1.35	32.00	13.21	54.79
3.8	0.31	50.74	3.30	0.30 ^b^	56.48	5.16 ^a^	72.56 ^abc^	10.95 ^b^	2.56 ^bc^	1.35	32.96	12.70	54.34
3.0	0.36	50.29	3.14	0.30 ^b^	55.24	4.75 ^a^	69.85 ^ab^	10.97 ^b^	2.33 ^abc^	1.34	33.19	12.63	54.18
3.4	0.36	49.87	3.16	0.30 ^b^	53.61	5.35 ^b^	74.85 ^bc^	10.83 ^b^	2.57 ^bc^	1.35	33.39	12.14	54.47
3.8	0.36	49.01	3.36	0.30 ^b^	53.53	4.82 ^a^	70.95 ^ab^	10.56 ^ab^	2.25 ^ab^	1.33	33.48	12.85	53.67
3.0	0.41	50.37	3.71	0.30 ^b^	53.15	5.96 ^b^	78.36 ^c^	12.15 ^d^	2.68 ^c^	1.37	32.92	12.72	54.36
3.4	0.41	50.68	3.37	0.28 ^a^	53.53	5.03 ^a^	72.09 ^ba^	10.18 ^a^	2.50 ^bc^	1.34	32.50	12.73	54.77
3.8	0.41	51.35	3.29	0.31 ^bc^	51.38	4.78 ^a^	69.89 ^ab^	11.61 ^cd^	2.33 ^abc^	1.34	32.97	13.37	53.66
SEM		0.279	0.056	0.002	0.450	0.089	0.682	0.063	0.043	0.005	0.002	0.001	0.414
Ca (%)	3.0	50.37	3.49	0.31	54.59	5.25	73.40	11.63 ^b^	2.53	1.34	32.47	12.59	54.89
	3.4	50.42	3.33	0.30	53.52	4.99	71.67	10.71 ^a^	2.34	1.35	32.57	12.70	54.68
	3.8	50.37	3.32	0.30	53.80	4.92	71.14	11.04 ^b^	2.38	1.34	33.09	12.97	53.89
	*p*-value	0.997	0.414	0.178	0.615	0.294	0.387	<0.001	0.198	0.913	0.211	0.339	0.213
NPP (%)	0.31	50.63	3.47	0.31	55.09	4.94	70.88	11.28 ^b^	2.36	1.34	32.10 ^a^	12.79	55.09
	0.36	49.72	3.22	0.30	54.13	4.97	71.88	10.79 ^a^	2.38	1.34	33.40 ^b^	12.52	54.10
	0.41	50.81	3.46	0.30	52.69	5.26	73.45	11.31 ^b^	2.50	1.35	32.80 ^ab^	12.95	54.26
	*p*-value	0.195	0.115	0.269	0.089	0.246	0.309	<0.001	0.321	0.285	0.010	0.286	0.866
Ca × NPP	*p*-value	0.687	0.327	0.049	0.330	0.005	0.006	<0.001	0.001	0.130	0.568	0.257	0.130

Note: Ca = calcium; NPP = non-phytate phosphorus; EW = egg weight; ESS = eggshell strength; EST = eggshell thickness; AH = albumen height; HU = Haugh unit; YC = yolk color; EG = egg grade; ESI = egg shape index; RYW = relative yolk weight; RESW = relative eggshell weight; RAW = relative albumen weight.

**Table 5:** Effects of dietary calcium and non-phytate phosphorus levels on serum lipid metabolism and antioxidant capability of 58-week-old *Beijing You Chicken* (*n* = 12).

Items		Serum Ca (mmol/L)	Serum P (mmol/L)	TG (mmol/L)	TC (mmol/L)	Glu (mmol/L)	HDL-C (mmol/L)	LDL-C (mmol/L)	MDA (nmol/mL)	T-AOC/(mmol/L)	LDH (U/L)
Ca (%)	NPP (%)										
3.0	0.31	1.75	1.46	11.37	2.26	11.88	2.98 ^a^	3.89 ^abc^	8.62	0.56	5914.88
3.4	0.31	1.81	1.28	17.84	3.00	9.75	3.08 ^a^	4.08 ^abc^	6.33	0.59	10,972.73
3.8	0.31	1.83	1.20	14.43	2.55	10.41	3.79 ^ab^	4.02 ^abc^	8.59	0.48	7685.12
3.0	0.36	1.87	1.48	11.91	3.16	13.16	4.00 ^ab^	4.97 ^bc^	6.44	0.64	7235.54
3.4	0.36	1.77	1.16	5.79	2.45	11.51	9.41 ^c^	3.37 ^ab^	5.58	0.62	8752.88
3.8	0.36	1.87	1.45	12.65	2.45	9.54	2.75 ^a^	3.19 ^ab^	13.41	0.58	8387.60
3.0	0.41	1.71	1.44	15.39	4.96	12.91	3.72 ^ab^	1.78 ^a^	23.06	0.49	8809.09
3.4	0.41	1.81	1.07	22.17	3.96	12.18	5.14 ^b^	6.24 ^c^	37.06	0.57	5971.07
3.8	0.41	1.82	1.91	13.39	2.85	12.78	4.86 ^b^	4.54 ^bc^	32.57	0.40	4762.81
SEM		0.018	0.076	1.963	0.642	0.441	0.213	0.416	2.587	0.057	511.534
Ca (%)	3.0	1.78	1.46	12.89	3.46	12.65	3.57	3.55	12.71	0.56	7319.84
	3.4	1.80	1.17	15.27	3.14	11.15	5.88	4.57	16.32	0.59	8565.56
	3.8	1.84	1.52	13.49	2.62	11.00	3.80	3.96	18.19	0.49	6945.18
	*p*-value	0.311	0.069	0.693	0.689	0.160	0.271	0.249	0.675	0.523	0.418
NPP (%)	0.31	1.80	1.31	14.55	2.60	10.68	3.28	3.99	7.85 ^a^	0.54	8190.91
	0.36	1.84	1.36	10.12	2.68	11.54	5.39	3.89	8.48 ^a^	0.61	8125.35
	0.41	1.78	1.47	16.98	3.92	12.62	4.57	4.19	30.90 ^b^	0.49	6514.32
	*p*-value	0.376	0.551	0.074	0.138	0.098	0.051	0.874	0.002	0.625	0.339
Ca × NPP	*p*-value	0.371	0.174	0.193	0.669	0.250	0.023	0.007	0.795	0.994	0.139

Note: Ca = Calcium; NPP= non-phytate phosphorus; TG = triglycerides; TC = total cholesterol; Glu = glucose; HDL-C = high-density lipoprotein cholesterol; LDL-C = low-density lipoprotein cholesterol; MDA = malondialdehyde; T-AOC = total antioxidant capacity.

**Table 6:** Effects of dietary calcium and non-phytate phosphorus levels on follicular development of 58-week-old *Beijing You Chicken* (*n* = 4).

Items		Number of Large Yellow Follicles	Number of Small Yellow Follicles	Number of Graded Follicles
Ca (%)	NPP (%)			
3.0	0.31	2.25 ± 2.63	16.00 ± 6.56 ^abc^	0.50 ± 0.58
3.4	0.31	2.25 ± 2.63	8.25 ± 8.18 ^a^	0
3.8	0.31	1.75 ± 2.06	9.50 ± 8.81 ^ab^	0.50 ± 0.58
3.0	0.36	0	10.00 ± 5.77 ^ab^	0.25 ± 0.50
3.4	0.36	1.00 ± 2.00	25.75 ± 4.34 ^c^	0
3.8	0.36	2.00 ± 2.45	25.00 ± 7.07 ^c^	0
3.0	0.41	3.00 ± 2.16	10.75 ± 7.09 ^ab^	0
3.4	0.41	1.50 ± 2.38	20.75 ± 3.50 ^bc^	0
3.8	0.41	3.75 ± 1.26	19.50 ± 5.45 ^abc^	0.25 ± 0.50
Ca (%)	3.0	1.75	12.25	0.25
	3.4	1.58	18.25	0
	3.8	2.50	18.00	0.25
	*p*-value	0.533	0.104	0.165
NPP (%)	0.31	2.08	11.25 ^a^	0.33
	0.36	1.00	20.25 ^b^	0.08
	0.41	2.75	17.00 ^b^	0.08
	*p*-value	0.141	0.021	0.165
Ca × NPP	*p*-value	0.536	0.033	0.443

Note: Ca = calcium; NPP = non-phytate phosphorus.

## Data Availability

None of the data were deposited in an official repository. The data that support the study findings are available upon reasonable request.

## Supplementary Materials

The following supporting information can be downloaded at: https://www.mdpi.com/article/10.3390/ani16193017/s1, Table S1: The catalogue numbers and assay methods for the commercial kits.
