# Supplementary material for: Effects of Dietary Calcium and Non-Phytate Phosphorus Interaction on Performance, Egg Quality and Lipid Metabolism of Aged Local Laying Hens
Source: Animals (Basel). 2026 Sep 25;16(19):3017. doi: 10.3390/ani16193017 (PMC13663330; doi:10.3390/ani16193017)
Supplement: Supplementary file 1 [file animals-16-03017-s001.zip › animals-4479078-supplementary.pdf]

Table S1. The catalogue numbers and assay methods for the commercial kits.

Parameter	Manufacturer	Catalogue No.	Assay Method
calcium	Nanjing Jiancheng	C004-2-1	Thymol blue method
phosphorus	Nanjing Jiancheng	C006-2-1	Phosphoric acid method
Triglycerides	Nanjing Jiancheng	A110-1-1	GPO-PAP enzymatic method
Total cholesterol	Nanjing Jiancheng	A111-1-1	COD-PAP method
High-density lipoprotein cholesterol	Nanjing Jiancheng	A112-1-1	Chemical modification method
Low-density lipoprotein cholesterol	Nanjing Jiancheng	A113-1-1	Enzyme colorimetric method
Lactate dehydrogenase	Nanjing Jiancheng	A020-2	Dinitrobenzaldehyde colorimetric method
Malondialdehyde	Nanjing Jiancheng	A003-1	TBA method
Total antioxidant capacity	Nanjing Jiancheng	A015-2-1	ABTS method
Glucose	Nanjing Jiancheng	A154-1-1	Glucose oxidase method
